# Morning report for all: a qualitative study of disseminating case conferences via podcasting

**DOI:** 10.1186/s12909-021-02799-1

**Published:** 2021-07-22

**Authors:** Gregory M. Ow, Lindsey C. Shipley, Saman Nematollahi, Geoffrey V. Stetson

**Affiliations:** 1 University of California, 513 Parnassus, Suite S-245, San Francisco, CA 94143-0454 USA; 2 University of Alabama, Birmingham, USA; 3 Johns Hopkins University, Baltimore, USA; 4 San Francisco VA Medical Center, San Francisco, USA

**Keywords:** Clinical reasoning, Diagnostic reasoning, Podcast, Technology enhanced learning, Case conference, Morning report, Medical education

## Abstract

**Background:**

Despite its long-established importance, diagnostic reasoning (DR) education has suffered uneven implementation in medical education. *The Clinical Problem Solvers* (*CPSolvers*) podcast has emerged as a novel strategy to help teach DR through case conferences with expert diagnosticians and trainees. *CPSolvers* has 25,000 listeners in 147 countries. The aim of this study was to evaluate the podcast by eliciting the developers’ goals of the podcast, then determining to what extent they aligned with the listeners’ actual usage habits, features they valued, and perceptions of the podcast.

**Methods:**

We conducted semi-structured interviews with 3 developers and 8 listeners from April–May 2020, followed by qualitative thematic analysis.

**Results:**

Three major developer goals with sub-goals resulted:

1. To teach diagnostic reasoning in a case-based format by (1a) teaching schemas, (1b) modeling expert diagnostic reasoning, (1c) teaching clinical knowledge, and (1d) teaching diagnostic reasoning terminology.
2. To change the culture of medicine by (2a) promoting diversity, (2b) modeling humility and promoting psychological safety, and (2c) creating a fun, casual way to learn.
3. To democratize the teaching of diagnostic reasoning by leveraging technology.

Listeners’ usage habits, valued features, and perceptions overall strongly aligned with all these aspects, except for (1c) clinical knowledge, and (1d) diagnostic reasoning terminology. Listeners identified (1a) schemas, and (2c) promotion of psychological safety as the most valuable features of the podcast.

**Conclusion:**

*CPSolvers* has been perceived as a highly effective and novel way to disseminate DR education in the form of case conferences, serving as an alternative to traditional in-person case conferences suspended during COVID-19. *CPSolvers* combines many known benefits of in-person case conferences with a compassionate and entertaining teaching style, plus advantages of the podcasting medium — democratizing morning report for listeners around the world.

**Supplementary Information:**

The online version contains supplementary material available at 10.1186/s12909-021-02799-1.

## Background

Diagnostic errors in medicine are one of the leading causes of death in the United States [1]. To help decrease diagnostic errors, the National Academies of Sciences issued a report that urged educators to improve clinical reasoning (CR) [2]. Additionally, there was a recent call to action for the ACGME (Accreditation Council for Graduate Medical Education) to include CR as a core competency [3]. However, despite its acknowledged importance, structured curricula for CR have suffered uneven implementation, with 57% of institutions in a national survey lacking such curricula [4]. Major barriers cited were lack of curricular time and faculty expertise, the former of which is likely exacerbated by the COVID-19 pandemic. Of the surveyed schools, the majority of CR education was provided during attending rounds (69%) or morning report (60%).

*The Clinical Problem Solvers* (*CPSolvers*) is a podcast with 25,000 listeners in 147 countries that may help fill this gap in CR education [5]. *CPSolvers* teaches diagnostic reasoning (DR), a component of CR which is the process by which clinicians cogitate to assign a label to a patient’s presentation [6]. *CPSolvers* is part of the emerging entity of engaging medical podcasts [7], a form of technology-enhanced learning (TEL) [8]. Podcasts and TEL have been widely adopted by trainees to provide asynchronous supplemental education [9–11], with calls to completely transition the undergraduate medical education pre-clerkship curriculum to TEL [12].

The *CPSolvers* podcast was launched in December 2018 by Rabih Geha, Sharmin Shekarchian, Arsalan Derakhshan, Daniel Minter, and Reza Manesh and has over 150 episodes to date. Each podcast episode is built around a case to simulate the diagnostic journey from initial patient encounter to final diagnosis. Cases are presented to expert diagnosticians and trainees in aliquots of clinical information, providing natural stopping points for discussion. Listeners are encouraged to think along with discussants. *CPSolvers* emphasizes teaching diagnostic schemas, conceptual frameworks that provide a structured approach to specific clinical problems [13, 14].

*CPSolvers* teaches and models DR in a case conference/morning report format, which in its in-person form, is highly valued by learners as a way to improve knowledge, problem-solving, and data gathering [15]. This study aimed to examine podcasting as a novel way to disseminate DR education in the form of case conferences by evaluating concordance between the developers’ goals and expectations of the podcast, compared to the listeners’ actual usage habits, features they valued, and perceptions of the podcast.

## Methods

As this study was interested in eliciting expectations and perceptions, a qualitative design was chosen. This design lends itself to the exploratory nature of the study, and evaluates to what extent listeners’ usage habits, valued features, and perceptions of the podcast aligned with the developers’ intentions. We utilized semi-structured interviews with a sample of developers and listeners, followed by thematic analysis. Data were collected from April to May 2020. This study was deemed exempt by the University of California, San Francisco’s Institutional Review Board.

### Sample and recruitment

Three *CPSolvers* developers (R.G., S.S., and R.M.) were recruited via email in April 2020 and were chosen based on diversity of viewpoints and time of involvement in the podcast. In April 2020, a subset of podcast listeners were recruited by announcing the study on an episode of *CPSolvers.* The study included all English-speaking podcast listeners who replied to our initial invitation. 11 podcast listeners (all English-speaking) replied, and all were offered interviews. Of these, 3 listeners were unavailable for interviews during our study period and were excluded. The remaining 8 listeners were available for interviews and participated in our study. All who were offered interviews received informed consent regarding the de-identification of their data. Those who were interviewed provided verbal consent at time of interview, were informed about the opportunity to stop the interview at any time, skip any questions, and were provided study contact information for follow up questions or to retain/redact data.

### Procedure

With expert guidance, G.O. developed semi-structured interview guides for developers and listeners (Supplemental Materials) based on existing evaluation instruments for TEL in medical education [8]. Interviews with the developers focused on eliciting their goals and expectations of the podcast, with questions like: “How did you design the podcast? What specific goals did you have? What aspects of the podcast do you think are most useful for listeners?” Interviews with the listeners focused on eliciting their usage habits, features valued, and perceptions of the podcast, with questions like: “How do you use the podcast? What do you find most valuable about the podcast? How would you describe the podcast to a colleague?” G. O. conducted and transcribed the 25–45 min video teleconference interviews from April to May 2020.

### Data analysis

Two authors (G.O., L.S.) independently analyzed all interview transcripts. We used a general qualitative approach with a constructivist orientation to generate themes [16, 17]. The initial codebook was created from the analysis of two developer interviews by G.O. These codes were then independently applied to the same two developer interviews by L.S. The initial codebook was then reviewed and revised by all authors, primarily to identify parent codes, collapse related codes, and confirm coding agreement. This codebook was then applied to the remaining developer interview, and a listener interview. A final codebook review was conducted by all authors to ensure they were in agreement, with minimal changes to the codebook. This final codebook was then used by G.O. and L.S. to independently code the remaining seven listener transcripts. All authors then reviewed the codebook and coded excerpts to generate the themes that described the developers’ goals and to what extent listeners’ interviews aligned with them. All authors had input on the representative quotes included in the Results section. Dedoose 8.3.35 (SocioCultural Research Consultants, LLC, Los Angeles, California) was used for analysis of the interview transcripts.

The following techniques were used to enhance rigor of the data analysis: G.O., who conducted all the interviews, also coded all of the transcripts and performed the data analysis; all transcripts were dual-coded by L.S.; and the codebook and results were reviewed by the entire research team.

### Researcher reflexivity

The research team consisted of physicians and a medical student. All interviews were conducted by the first author G.O., a 4th year medical student at the time of interview. His experience as a medical student and as a podcast listener may have influenced the collection and interpretation of data; it is possible that interviewees who were medical students were more comfortable talking with him because of their shared training stage, or that interviewees of more advanced training stage were less comfortable due to their different training stages. During the analysis, other members of the team, L.S. and S.N., who are on the *CPSolvers* team, contributed additional perspectives to that of the first author. Their additional internal knowledge of the podcast may have influenced their interpretation of the data. The study supervisor G.S., who is a physician and medical educator and has conducted prior research related to technology in medical education, contributed additional perspectives and guided overall data interpretation. His additional a priori knowledge may have influenced this study’s interpretation of the data.

## Results

A total of 3 developers and 8 listeners were interviewed. As shown in Table 1, half of the listener participants were female (*n* = 4), and the majority were medical students (*n* = 6).

**Table 1:** Sample Selected Characteristics (*n* = 8)

Characteristic	Listener Interviewees (n, (%))
*Demographic*	
Female	4 (50)
Male	4 (50)
*Age*	
25–30	6 (75)
31–35	2 (25)
*Training Stage*	
MS3 - Pre-clerkship	1 (12.5)
MS3 - Post-clerkship	4 (50)
MS4	1 (matched into internal medicine, 12.5)
PGY-1	1 (internal medicine, 12.5)
PGY-2	1 (OB-GYN, 12.5)
*Institutions Represented*	
	Emory University Massachusetts General Hospital New York University St. Louis University Tufts University University of California, San Francisco University of Texas at Austin University of Washington

MS3 - third year medical student. MS4 - fourth year medical student. PGY - post-graduate year in residency

Results yielded three major goals for the developers: to teach diagnostic reasoning, to change the culture of medicine, and to technologically advance and democratize diagnostic reasoning education (Table 2). Each major goal had multiple sub-goals. When compared to the listener interviews, there was exceptionally strong alignment with the developers’ major goals, with the exception of two sub-goals regarding the teaching of diagnostic reasoning (1c and 1d, below). A detailed comparison is presented. All quotes are contextualized by a de-identified listener ID (e.g. Listener 1), or a developer ID (e.g. Developer 2).

**Table 2:** Themes: Developer Goals vs Listener Experiences

Developer Goals and Sub-Goals	Did Listener Interviews Align?
**(1)** To teach diagnostic reasoning in a case-based format by: **(1a)** teaching schemas **(1b)** modeling expert diagnostic reasoning **(1c)** teaching clinical knowledge **(1d)** teaching diagnostic reasoning terminology.	**(1a)** YES. Listeners cited schemas as the most useful feature of the podcast. **(1b)** YES. Listeners used the expert’s thinking to guide their own reasoning. **(1c) NO.** Listeners had enough ways to learn clinical knowledge, and instead struggle more with how to organize their existing knowledge. **(1d) NO.** 7 of the 8 listeners interviewed could not define DR terminology used in the podcast.
**(2)** To change the culture of medicine by: **(2a)** promoting diversity **(2b)** modeling humility and promoting psychological safety **(2c)** creating a fun, casual way to learn.	**(2a)** YES. Listeners felt the diversity of participants in the podcast led to a greater sense of belonging. **(2b)** YES. Listeners found normalization of the learning process to be psychologically freeing, resulting in better learning. **(2c)** YES. Listeners perceived the podcast as an entertaining way to learn, with time constraints leading to a more casual use of the podcast.
**(3)** To democratize the teaching of diagnostic reasoning by leveraging technology.	**(3)** YES. Listeners perceived the podcast as a novel way to learn DR, helping to fill gaps in their education.

### Developer goal 1: to teach diagnostic reasoning in a case-based format by (1a) teaching schemas, (1b) modeling expert diagnostic reasoning, (1c) teaching clinical knowledge, and (1d) teaching diagnostic reasoning terminology

The developers described aiming to improve listeners DR through several different ways. These included teaching more declarative knowledge such as (1c) clinical knowledge and (1d) DR terminology, and procedural knowledge such as (1a) teaching approaches to specific clinical problems and (1b) modeling the diagnostic reasoning process itself.

#### (1a) Teaching Schemas

When asked to describe the most useful feature of their podcast, all the developers thought the diagnostic schemas were by far the most valuable, because listeners could immediately apply the frameworks and approaches to their daily practice. The podcast developers all expressed the specific goal of wanting to disseminate their diagnostic schemas to the world. The *CPSolvers* developers perceived schemas as diagnostic reasoning tools from experts that, if shared widely, would help to elevate every doctor’s reasoning:

*We know all of these incredible practices, and what expert diagnosticians do. So how can we apply that to every single doctor?... Schemas are a very practical way to do that.* (Developer 2)

The listener experiences strongly aligned with this goal. Most listeners reported that the schemas were the most useful things they took away from the podcast, and all listeners thought that the schemas were highly useful, stating:

*[Schemas] give me the power to be able to think [like an expert] when I see a patient.* (Listener 1)

Listeners saw learning schemas in the same way as developers: as a way to bring expert diagnostic reasoning into their own patient encounters.

#### (1b) Modeling Expert Diagnostic Reasoning

Developers of *CPSolvers* wanted to teach the reasoning processes of master diagnosticians. To do this, the developers wanted to provide expert discussion on the podcast which would prompt listeners to compare and contrast their own thinking with that of the expert’s:

*How do you model reasoning? How do you model vulnerability when you’re uncertain?... People can think about their own reasoning and then compare it to whatever an expert does.* (Developer 2)

Listeners’ description of what they valued in the podcast aligned with this goal. They specifically stated that they use the expert’s thinking to guide their own reasoning:

*The best way I sort of test my reasoning skills is by referencing it to those who are more experienced than I am.* (Listener 5)

However, while the developers robustly described their own process of solving a case as deliberately trying to create a simulative experience, the listeners struggled to articulate their process.

*[When I solve a case] it’s really a simulation, the same way basketball players have a practice game. You know, they practice and then they play games that count. It was trying to create that atmosphere, that simulation for us to have our practice.* (Developer 3)

Even as listeners stated that they were comparing their reasoning with the expert’s reasoning, they could not describe how they accomplished this cognitive task, nor could they describe tangible lessons they learned from doing this.

*I pretty much feel like I’m just in an absorbing phase of just trying to figure out my own way of thinking, so I just try to follow along like, ‘oh, yeah, that makes sense.’* (Listener 4)

The listeners’ approach to comparing their reasoning with the expert’s was far less intentional than the developers’ approaches.

#### (1c) Teaching Clinical Knowledge

The developers stated that the foundation of diagnostic reasoning expertise is a robust understanding of disease presentations and pathophysiology:

*A deeper mastery of the topic itself provides clarity that that cannot be achieved in any other way... Clinical knowledge is queen.* (Developer 1)

As a result, the developers intended to teach and expected listeners to value learning clinical knowledge from the podcast.

However, listeners actually placed relatively little value on learning facts about specific diseases. Listeners stated that they had enough ways to learn clinical knowledge from classes, clinical experiences, and conferences, but struggled more with how to organize their existing knowledge:

*I have had scattered information from different classes and courses; I had never had a course that consolidated all of this [like this podcast does]* (Listener 1)

Instead of listening to the podcast to learn new clinical knowledge, listeners actually primarily listened to the podcast to learn schemas to organize their existing knowledge (1a, above).

#### (1d) Teaching Diagnostic Reasoning Terminology

The developers stated that they wanted to deliberately use the formal terminology of diagnostic reasoning in order to more quickly advance listener’s progress. The developers expected that listeners would be learning the terminology given how frequently they used them.

*If you’re able to teach these concepts [diagnostic reasoning terminology], then you expedite the learner’s ability to understand the importance of how you frame a case... we try to use those as frequently as we can to give learners these tools (Developer 3)*

However, despite this intentional use by the developers, only one listener discussed this idea as a benefit of the podcast. When prompted, 7 of the 8 listener interviewees were unable to define common terminology used throughout the podcast such as “problem representation” or “illness script.” The listeners noted that they heard these phrases repeatedly on each episode, but were not clear on what the terms actually meant.

### Developer goal 2: to change the culture of medicine by (2a) promoting diversity, (2b) modeling humility and promoting psychological safety, and (2c) creating a fun, casual way to learn

In addition to teaching diagnostic reasoning (Goal 1), the developers also sought to use their podcast as a platform to change the culture of medicine. The developers presented a new vision of medical education, one that was diverse, compassionate, and fun.

#### (2a) Promoting Diversity

The *CPSolvers* team has known that the field of diagnostic reasoning has primarily featured white men. So to promote diversity, a guiding principle of the podcast was to include women, and non-white people as participants in the discussion. Beyond personal demographics, *CPSolvers* developers also wanted to feature physicians and learners from institutions that are typically underrepresented in medical education discourse:

*[To] give voice to people who sometimes are fearful of expressing it... to eliminate shame in medicine... to bring in through the ranks women, people who identify as minorities in medicine... to bring people from all over the world together to get better diagnoses.* (Developer 1)

Listeners all appreciated the diversity of participants in the podcast:

*There’s a real variety of people of different representations... they’re doing a great job to try to diversify the representation in medical education.* (Listener 5)

Listeners felt this diversity of representation created a greater sense of belonging and a safe learning environment.

#### (2b) Modeling Humility and Promoting Psychological Safety

The developers felt that the culture of medicine too often prioritized perfectionism at the expense of life-long learning, resulting in trainees feeling shame as they grappled with their fallibility. To change this, the developers wanted to instead model a humble, compassionate approach to medicine by exposing their own vulnerabilities. The developers wanted to show learners that even attending physicians made and make mistakes, that even expert diagnosticians have gaps in their knowledge:

*[The podcast] is an incredibly vulnerable place to think out loud, right? The humility that comes from it, the whole concept of we’re all learners. None of us are perfect, that we all make mistakes, and how do you learn from it? I think that’s what’s very much lacking in this space in general. I mean depending on where you do medicine or residency, this whole notion that doctors are supposed to know everything. No that’s not the case! And I feel very proud of us for being like ‘I don’t know, I’ll look it up!’* (Developer 2)

Listeners expressed relief from hearing these expert and eloquent physicians reveal gaps in their knowledge or times when they made mistakes. The listeners felt that this process normalized the learning process, humanized the expert discussants, and demonstrated that there is a continuum of growth on which all doctors lie:

*[Acknowledging mistakes] normalizes the fact that everyone is learning, even though some people are further along than others.* (Listener 2)

In addition to modeling a humble approach to experts’ mistakes, the developers also wanted to create a learning environment where learners could freely expose their knowledge gaps without fear of humiliation or shame. The developers wanted to promote psychological safety in order to create better learning:

*If you’re asking a person to be vulnerable, to share their thinking, to expose their gaps — you better create an environment [in which] that’s okay, and also teach them the knowledge gap and inspire them to keep learning.* (Developer 2)

This approach strongly resonated with the listeners:

*Saying ‘I have no idea,’ and for them to say ‘Yes, I love to hear that’ [is incredible.] Because I feel like you really do learn a lot more when you get things wrong.* (Listener 4)

All listeners interviewed cited this compassionate approach as not only psychologically freeing, but also improving their learning.

#### (2c) Creating a Fun, Casual Way to Learn

The developers noted that listeners have many other things competing for their attention, let alone other podcasts. As such, they wanted to hold listener’s attention by making the podcast fun:

*[We’re trying to make] the experience [of listening] not just a ‘Can I learn from this?’ but ‘I actually had fun.’* (Developer 2)

This emphasis on fun carried over to the listener experience. While none of the listeners interviewed used the podcast for entertainment, they all perceived the podcast as an entertaining way to learn — a way to “study, but not be studying”:

*[The podcast is] not like ‘I have to study or listen to this lecture.’ It’s something that I do with an open heart. I love doing it. It’s pleasant to me.* (Listener 1)

As a result of the fun nature of the podcast, the listeners felt more intrinsic motivation to listen to the podcast.

However, even as they intentionally made the podcast fun to hold listener attention, the developers were forced to contend with their expectation that listeners would treat the podcast as a casual activity, resulting in suboptimal learning. For example, when asked to describe the ideal way to listen to the podcast, the developers described a serious and dedicated listening habit:

*[The ideal way to listen would be] in a quiet space, you might have some candles on... one aliquot is said, you pause, you write down all of your thoughts for five minutes, click play, compare your thinking.* (Developer 3)

Yet when asked to guess what proportion of listeners actually pause episodes to think for themselves, the guesses were 4, 7.5, and 30%, demonstrating that the developers thought the vast majority of listeners were not engaging in these high-effort but high-yield activities.

But instead of trying to push the listeners into these habits, the developers recognized that the podcast would be more effective if they just acknowledged the casual nature in which podcasts are used. When asked if they would implement forced pauses in the podcast:

*We tried for just 3 s one time and the listeners hated it — absolutely hated it! I think if we did that, no one would listen to us!* (Developer 1)

This intuitive and responsive approach aligned well with the listener usage habits. All listeners described dedicated listening sessions with pausing as the best way to maximize learning, but cited time constraints as rendering best practices infeasible:

*[The ideal way to listen would be] You should sit down, and take notes, and if you don’t understand something, pause, rewind, think about it... but I think just with time limitations, I don’t know if that’s feasible.* (Listener 6)

Of the listeners interviewed, no listener had a specific method for how they listened to the podcast, all of them report essentially never pausing podcasts to think, and only one of them ever, albeit rarely, had dedicated listening sessions:

*[When I listen,] it’s usually not in a setting where I’m writing down something, so you know, working out, cooking, maybe taking a walk.* (Listener 5)

Rather than sitting down to study the podcast, listeners instead used the podcast to overlay education in an entertaining way onto “downtime.”

### Developer goal 3: to democratize the teaching of diagnostic reasoning by leveraging technology

The developers recognized that the landscape of medical education was changing — especially with the COVID-19 pandemic — and they sought to leverage podcasting technology to make diagnostic reasoning education accessible for everyone:

*Medical education is changing, the landscape is changing, and I think the COVID pandemic has only accelerated what was inevitable, whether it be Zoom teaching, whether it be podcast, whether it be Twitter, and we were trying to be part of that technology and be at that forefront of medical education... everyone can have access to our recording — but they can listen to it driving to work, running on the streets, or even it could be used as like a lecture in a class.* (Developer 3)

The developers identified several advantages of the podcasting medium: the ability to listen while multitasking, the possibility of listening to episodes multiple times, and the ability to pause an episode in order to think through a case before proceeding.

This aligned strongly with listeners’ perceptions of the podcast. Listeners thought that the podcast was a novel way to learn diagnostic reasoning. Although they did not perceive it as ideal learning (see 2c, above), the listeners pointed to the ability to multitask as a benefit of the medium. Lastly, the listeners saw the podcast as filling in gaps in their medical education, especially morning report:

*We don’t really have morning reports, which has been one of the things that I’ve actually loved about having CPSolvers.* (Listener 2)

Listeners from institutions that do not have regular case conferences, or from institutions where morning report was suspended during the COVID-19 pandemic greatly valued continued access to case-based clinical learning.

## Discussion

This study demonstrates that the three goals of the *CPSolvers* podcast — teaching diagnostic reasoning, changing the culture of medicine, and democratizing DR education — overall strongly aligned with listeners’ usage habits, valued features, and perceptions, with two exceptions.

### Goal 1: to teach diagnostic reasoning

The developers wanted to teach diagnostic reasoning via four different avenues, and listeners strongly resonated with two of them: (1a) schemas, and (1b) modeling expert DR. The listeners placed heavy value on these features specifically because of their scarcity; the listeners felt that these provided effective learning and could not otherwise be found in their curricula. Conversely, the only gaps in developer expectation were around the listener experience of (1c) clinical knowledge and (1d) DR terminology. While the developers thought these two aspects served as a critical foundation to build DR expertise, the listeners felt that they received plenty of clinical knowledge elsewhere in their curricula, and were not learning the DR terminology through the podcast. Collectively, these findings suggest that listeners seek directly applicable general knowledge like schemas (which immediately help to organize knowledge), rather than ambiguously useful specific knowledge like details of individual disease entities or DR terminology (which in the long-term improve the metacognition of DR).

In addition, regarding (1b) modeling expert DR specifically, the developers’ approach of providing expert discussion for listeners to compare their thought processes is consistent with cognitive modeling [18], a component of cognitive apprenticeship [19] whereby learners internalize expert thought patterns through observation. *CPSolvers’* deliberate use of modeling in a reflective manner by explaining their rationale and process is one of the most effective ways to utilize cognitive modeling [19].

### Goal 2: to change the culture of medicine

The developers wanted to change the culture of medicine in three different ways, and listeners strongly resonated with all of them: (2a) promoting diversity, (2b) promoting psychological safety, and (2c) creating a fun/casual way to learn. Listeners report that this learner-centric approach improves their learning while also contributing to their well-being, yet note that this approach is too rarely found [20]. These results align with the broader literature of psychological safety in medical education, whereby students who do not feel judged have high psychological safety and are free to focus on learning in the present moment [21, 22]. Universal adoption of the *CPSolvers’* method of demonstrating compassionate support of learners and diversity would take great strides towards improving medical education for all.

In addition, regarding (2c) creating a fun/casual way to learn in particular, the listener’s expected use of the podcast while multitasking suggests *CPSolvers’* approach of assuming casual, distracted listeners is correct, confirming the necessity of frequent reorientation to the case/topic, repetition, and summary of key points in medical education podcasts [9, 23]. Notably, although the *CPSolvers* developers acknowledge the superior learning that would result from forced pausing and deliberate practice [24], they acknowledge that their listeners disliked it and the developers feared this would disenchant their audience.

### Goal 3: to democratize the teaching of diagnostic reasoning

The developers saw that the landscape of medical education was changing, especially during the COVID-19 pandemic where traditional in-person case conferences have been suspended. The developers wanted to leverage technology to fill this void and democratize the teaching of diagnostic reasoning. Listeners strongly resonated with this goal, expressing gratefulness that the podcast could replace their suspended in-person morning reports due to COVID-19, or could fill the gap since their institution at baseline did not have morning report. Additionally, both the developers and listeners cite the advantages of podcasting as audio-only asynchronous TEL, allowing listeners around the world to multitask, listen on-demand, and pause/repeat even during a pandemic.

### Overall

Thus when compared to in-person case conferences/morning report, *CPSolvers* appears to retain many of its known benefits, including providing case-based teaching, highlighting the unique approach of the generalist physician, and developing intellectual curiosity [15]. *CPSolvers* also appears to make several improvements in often-criticized aspects of in-person morning report including improving tone and providing a psychologically safe learning environment [15]. However, in-person case conferences also retain unique advantages: as compared to podcasts where multitasking is prevalent and expected, physical co-location likely encourages trainees to treat it as a dedicated activity, along with valued opportunities for socialization [15, 25].

Further investigation would provide valuable insight by comparing in-person conferences, with new asynchronous podcasts, with the even newer entity of synchronous video conferences [26]. In order to optimize DR education, each respective modality should specialize in their unique advantages for trainees (e.g. podcasts may be uniquely effective in transmitting “think aloud”-type content [27–29]). Each respective modality, especially upon the resumption of in-person case conferences, should also seek to apply lessons learned from the other modalities (e.g. promoting diversity and psychological safety like the *CPSolvers* podcast during in-person conferences).

### Limitations

This study had a limited number of interviewees from a much larger group of listeners. Thus these findings have limited generalizability and offer some initial early insights into how the developer goals align with listener usage habits, features values, and perceptions [30]. Next steps include further exploration of usage and efficacy with a quantitative survey among a larger sample of participants.

## Conclusion

Overall, this evaluation concludes that *CPSolvers* has been perceived as a highly effective and novel way to disseminate DR education in the form of case conferences, serving as an alternative to traditional in-person case conferences suspended during COVID-19. This study characterizes the goals of the *CPSolvers* developers and demonstrates that listeners’ usage habits, valued features, and perceptions align strongly with those goals. Further research is needed to explore if these findings are generalizable in a larger sample of participants and to compare asynchronous case-discussions to synchronous in-person and video-based case-conferences.

## Supplementary Information

**Additional file 1.** Supplemental Materials. Developer Interview Guide and Listener Interview Guide.

## Data Availability

The datasets generated and/or analysed during the current study are not publicly available due to interviewee confidentiality but are available from the corresponding author on reasonable request.

## Abbreviations

ACGME: Accreditation Council for Graduate Medical Education
CPSolvers: Clinical Problem Solvers
CR: Clinical reasoning
DR: Diagnostic reasoning
TEL: Technology-enhanced learning
