# Supplementary material for: Morning report for all: a qualitative study of disseminating case conferences via podcasting
Source: BMC Med Educ. 2021 Jul 22;21:392. doi: 10.1186/s12909-021-02799-1 (PMC8295545; doi:10.1186/s12909-021-02799-1)
Supplement: Supplementary file 1 — Additional file 1. Supplemental Materials. Developer Interview Guide and Listener Interview Guide. [file 12909_2021_2799_MOESM1_ESM.zip › Supplemental - Developer Exploratory InterviewsR1.docx]

# Developer Exploratory Interviews - Instrument

**OPEN-ENDED QUESTIONS**

1. Describe *The Clinical Problem Solvers* in your own words.
2. Can you tell me about how the podcast was designed?
   1. What do you think is effective about this format/design?
   2. What specific goals/or learning objectives did you have, if any?
   3. What features of the tool are designed specifically to meet their objectives?
3. What segments/aspects of the podcast do you think are most useful for listeners?
   1. How did you decide on that aspect?
4. Were you thinking about the podcast as a tool to support clinical reasoning?
   1. What did you do to support this goal?
   2. How have you changed the podcast over time to better support this goal?
5. What do you envision as optimal usage of the podcast? How often and in what ways?
   1. In what ways do you think listeners are actually using your podcast?
6. In what ways do you think this podcast is useful or helpful to listeners?
7. How do you think this podcast might enhance listeners’ clinical reasoning?

**PROBING QUESTIONS**

1. The podcast asks listeners to pause after each aliquot and think before the expert discussion starts. How did this idea originate?
2. Tell me about how the expert discussion format was designed.
   1. The expert discussion format follows several recommendations for clinical reasoning instruction, including the use of compare/contrast. How did this arise?
3. Tell me about the idea of combination of visual and auditory resources for the diagnostic schema. How did this arise?
