# Supplementary material for: Morning report for all: a qualitative study of disseminating case conferences via podcasting
Source: BMC Med Educ. 2021 Jul 22;21:392. doi: 10.1186/s12909-021-02799-1 (PMC8295545; doi:10.1186/s12909-021-02799-1)
Supplement: Supplementary file 1 — Additional file 1. Supplemental Materials. Developer Interview Guide and Listener Interview Guide. [file 12909_2021_2799_MOESM1_ESM.zip › Supplemental - Listener Exploratory InterviewsR1.docx]

# Listener Exploratory Interviews - Instrument

**OPEN-ENDED QUESTIONS**

1. **Cross-Compare Resources:**What resources do you use to support your development of clinical reasoning skills?
   1. What resources does your institution offer to develop clinical skills?
      1. Does your institution offer a case conference / M&M / morning report?
   2. (For the listed resources) How do you use it? What do you value in it?
2. **Usage Habits:**How do you use *The Clinical Problem Solvers* podcast?
   1. When do you use it? #/week
   2. Can you tell me how it looks when you are listening?
   3. Do you pause and think through the case?
   4. Do you jot down schema, look up articles or guidelines or other resources during or after? Access the CPS website or app?
   5. How do you use the expert commentary?
   6. Clinical Unknowns vs Schema episodes
   7. What do you think the ideal way to use the podcast is?
3. **Valued Features:**How would you describe the effect of this podcast on your clinical reasoning skills?
   1. What do you think you are gaining from it?
   2. Can you tell me a specific example that shows its impact on you?
   3. How is this tool similar or different to other tools that you use?
   4. What in particular about The Clinical Problem Solvers is most useful to you?
   5. What do you find most valuable about The Clinical Problem Solvers?
   6. What keeps you coming back to this resource?
4. **Perception:**
   How would you describe *The Clinical Problem Solvers* to a classmate?

Why should they listen to it?

- 1. Do you think of the podcast as entertainment or training, or both, or something else?
  2. Overall, what elements of this podcast have been most useful to you as a listener?

1. **Feedback:**What feedback do you have for *The Clinical Problem Solvers?*
   1. What would you add?
   2. Is there anything you would take away?
   3. What would you keep the same?
   4. What could be done to improve your engagement?
2. Is there anything else you'd like to share about your experience with *The Clinical Problem Solvers?*

Re: other podcasts, I'll incorporate some questions when I ask in general what other resources they use --- great suggestion.

Re: CPS schema: I'll adjust question 4e to ask more directly if they actually download/use any of the visual references. Thanks!

Re: the more detailed questions, I'll establish that data when I ask about their usage habits, but those will be ideal for the quantitative survey we'll generate.

- depending on the angle you are trying to take, can ask if they listen to other podcasts (curbsiders, CoreIM, cardionerds, etc) and ask them to rank or compare/contrast them to CPS

- can consider asking, do you use the CPS schema and/or illness script while listening?

- may be getting into more details than needed, but some of these questions may be useful to know because you may see a trend in trainee answers that listen to all the episodes vs some (as an example): how long have you been using CPS? how many times a week do you listen? have you listened to all the episodes? have you listened to some episodes more than once (if so, why?)
